# Developing High-Energy, Stable All-Solid-State Lithium Batteries Using Aluminum-Based Anodes and High-Nickel Cathodes

**DOI:** 10.1007/s40820-025-01751-y

**Published:** 2025-04-29

**Authors:** Xin Wu, Meiyu Wang, Hui Pan, Xinyi Sun, Shaochun Tang, Haoshen Zhou, Ping He

**Affiliations:** 1 Center of Energy Storage Materials & Technology, Department of Energy Science and Engineering, College of Engineering and Applied Sciences, Jiangsu Key Laboratory of Artificial Functional Materials, National Laboratory of Solid-State Microstructures, and Collaborative Innovation Center of Advanced Microstructures, Nanjing University, Nanjing, 210093 People’s Republic of China; 2 Department of Materials Science and Engineering, College of Engineering and Applied Sciences, Jiangsu Key Laboratory of Artificial Functional Materials, National Laboratory of Solid-State Microstructures, and Collaborative Innovation Center of Advanced Microstructures, Nanjing University, Nanjing, 210093 People’s Republic of China

**Keywords:** All-solid-state lithium battery, Ni-rich cathode, Pre-lithiated Al anode, High energy density, Interface modification

## Abstract

Aluminum (Al) exhibits excellent electrical conductivity, mechanical ductility, and good chemical compatibility with high-ionic-conductivity electrolytes. This makes it more suitable as an anode material for all-solid-state lithium batteries (ASSLBs) compared to the overly reactive metallic lithium anode and the mechanically weak silicon anode. This study finds that the pre-lithiated Al anode demonstrates outstanding interfacial stability with the Li_6_PS_5_Cl (LPSCl) electrolyte, maintaining stable cycling for over 1200 h under conditions of deep charge–discharge. This paper combines the pre-lithiated Al anode with a high-nickel cathode, LiNi_0.8_Co_0.1_Mn_0.1_O_2_, paired with the highly ionic conductive LPSCl electrolyte, to design an ASSLB with high energy density and stability. Using anode pre-lithiation techniques, along with dual-reinforcement technology between the electrolyte and the cathode active material, the ASSLB achieves stable cycling for 1000 cycles at a 0.2C rate, with a capacity retention rate of up to 82.2%. At a critical negative-to-positive ratio of 1.1, the battery’s specific energy reaches up to 375 Wh kg^−1^, and it maintains over 85.9% of its capacity after 100 charge–discharge cycles. This work provides a new approach and an excellent solution for developing low-cost, high-stability all-solid-state batteries.

**Supplementary Information:**

The online version contains supplementary material available at 10.1007/s40820-025-01751-y.

## Highlights

- Anode pre-lithiation technique was employed to promote the reversibility of Al.
- Dual-reinforcement technology was developed to address the interfacial incompatibility between the Ni-rich cathode active material and sulfide solid-state electrolyte.
- The fabricated all-solid-state lithium battery comprising the pre-lithiated Al anode and dual-reinforced Ni-rich cathode achieves stable cycling for 1000 cycles with a capacity retention of 82.2%.

**Supplementary Information:**

The online version contains supplementary material available at 10.1007/s40820-025-01751-y.

## Graphical abstract

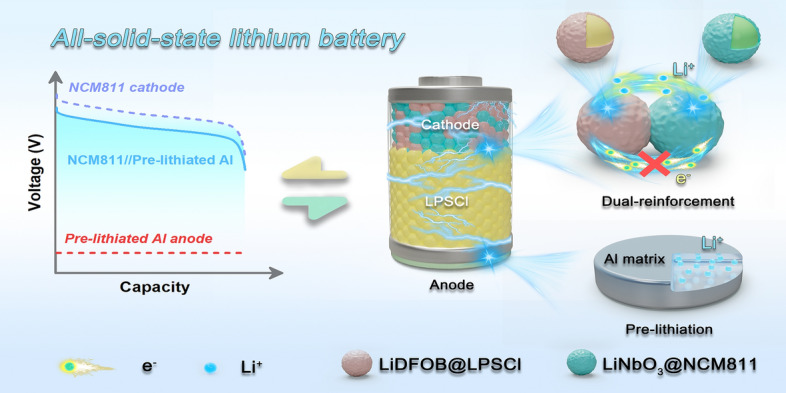

## Introduction

Sulfide-based all-solid-state lithium batteries (ASSLBs) with high energy density and safety are expected to satisfy the demands of long-range electric vehicles and electric flight [1]. As one of the key components of the battery, negative electrode plays a critical role in battery performance [2, 3]. Lithium (Li) metal anode, which exhibits low electrode potential (− 3.04 V vs. standard hydrogen electrode) and high theoretical capacity (3860 mAh g^−1^), has been extensively investigated for ASSLBs [4]. Nonetheless, the issues related to interfacial instabilities between Li and sulfide electrolytes, as well as the short circuits caused by Li dendrites penetrating the electrolyte, have proved to be exceedingly challenging to address [5–7].

Other alternative anode materials such as Li alloys not only retain a significant capacity advantage, but also possess improved interfacial stability due to the reduced thermodynamic driving force for electrolyte reduction. Moreover, Li alloys can promote uniform plating and stripping of Li^+^ and thus prevent the safety hazards caused by the growth of Li dendrites [6]. Metal indium (In) is a commonly used reversible counter electrode tool in the study of cathodes for sulfide-based ASSLBs. Nevertheless, its high operating potential (0.62 V vs. Li/Li^+^) and small electrochemical capacity hinder it from being utilized as an actual battery anode [8, 9]. High-capacity silicon (Si) materials also encounter extremely challenging difficulties in all-solid-state batteries. The chemical instability at the interface with the electrolyte and the stress failure due to significant volume deformation are both concerning issues [10, 11]. In addition, the poor conductivity of Si also raises concerns of slow kinetics during charge and discharge period.

Aluminum (Al), as the most abundant metallic element in the earth’s crust, has a good conductivity and competitive capacity of 990 mAh g^−1^. Compared with Si anode, Al exhibits smaller volume changes during cycling (96% vs. 320%), which is favorable for maintaining good anode–electrolyte interface stability. Moreover, the moderate working potential of Li-Al alloy (~ 0.3 V vs. Li/Li^+^) can also facilitate the realization of high energy density in batteries. Since Al can be economically and efficiently fabricated as a freestanding foil, the application of Al does not involve any inactive conductive agents and binders, nor require an additional current collector [12]. In consideration of these advantages, Al is a promising candidate for advanced anode material.

In our previous work, we proposed a stable all-solid-state lithium–sulfur battery system, in which Li_0.8_Al alloy was employed to replace Li as an anode to achieve excellent stability with the Li_10_GeP_2_S_12_ electrolyte [13]. However, the overall energy density of the battery is limited to the low operating potential of the S cathode (~ 2.0 V). High-nickel layered oxide cathode (LiNi_*x*_Co_*y*_Mn_1−*x*−*y*_O_2_, x ≥ 0.8, NCM), which delivers an output voltage of ~ 3.7 V and decent specific capacity, is desirable to enable a more practical ASSLB. Nevertheless, constrained by the severe interface incompatibility between the cathode active materials (CAMs) and sulfide electrolytes, the realization of stable sulfide-based all-solid-state battery composed of Al-based anode and high-nickel cathode is still impeded.

**Fig. 1:**
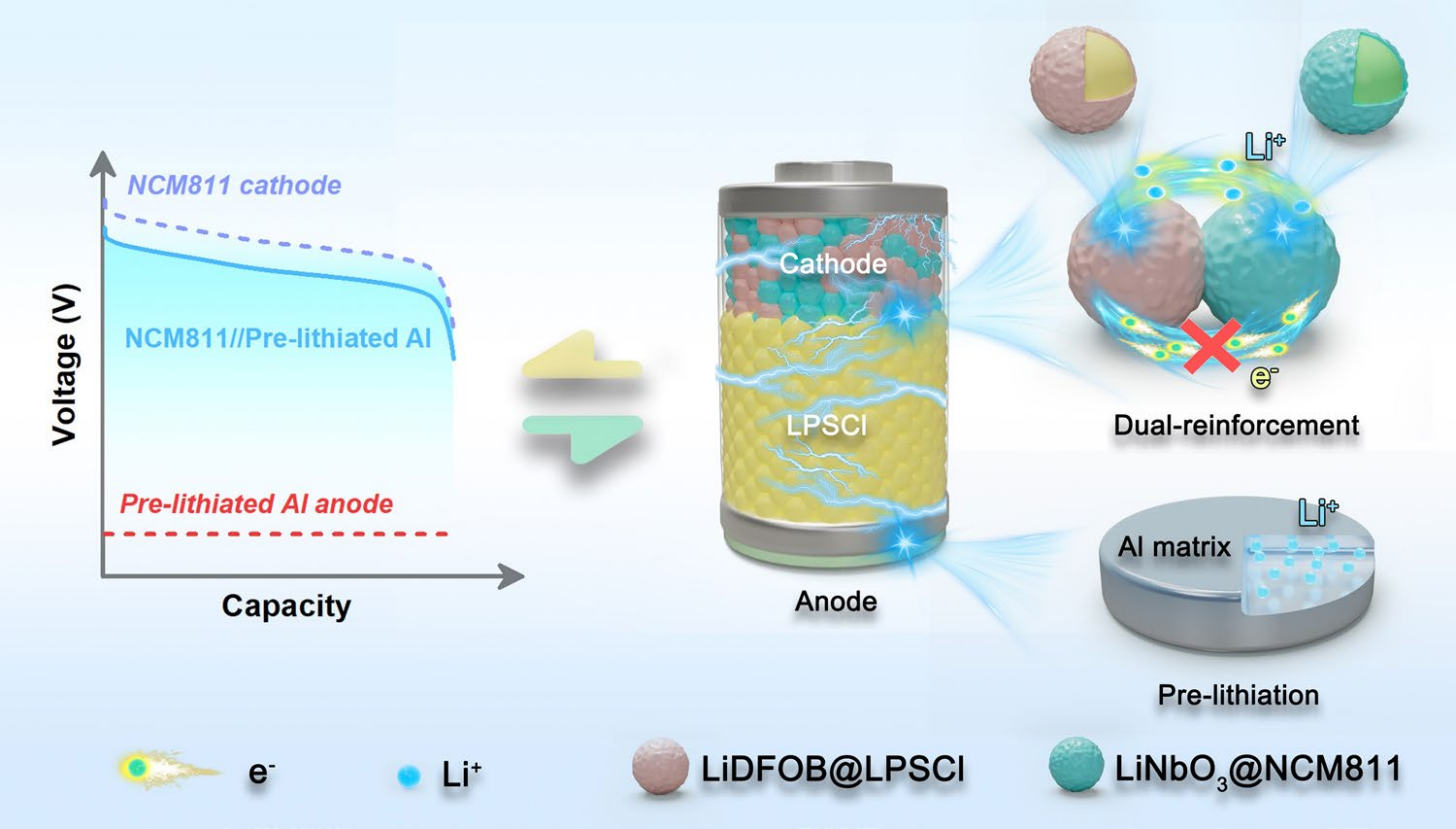
Schematic diagram of all-solid-state battery configuration composed of pre-lithiated Al anode and dual-reinforced NCM811 cathode

Here, by addressing the interface issues between the electrode materials and sulfide electrolytes, a promising type of ASSLB with high energy and stability was designed (as illustrated in Fig. 1). First, an anode pre-lithiation technique was adopted for Al to promote its reversibility, and the outstanding stability of the pre-lithiated Al anode toward sulfide electrolyte (Li_6_PS_5_Cl, LPSCl) was confirmed by the electrochemical tests of symmetric cells under deep charge–discharge conditions. Then, a dual-reinforcement technology was developed to effectively suppress the side reactions between the LiNi_0.8_Co_0.1_Mn_0.1_O_2_ (NCM811) CAMs and LPSCl electrolyte, as well as strengthen the oxidation tolerance of LPSCl at high potentials. The superior electrochemical properties of the modified composite cathode were identified by the cycling and rate tests, as well as the postmortem characterizations. Furthermore, an ASSLB composed of the well-designed negative and positive electrodes was fabricated and evaluated to verify the feasibility of the proposed battery system. The ASSLB achieved stable cycling for 1000 cycles with an excellent capacity retention of 82.2%. At a critical negative-to-positive (N/P) ratio of 1.1, the battery’s specific energy reaches up to 375 Wh kg^−1^ (based on the mass of the positive and negative electrodes), and it maintains over 85.9% of its capacity after 100 charge–discharge cycles, which demonstrates a bright practical prospect of the ASSLB.

## Experimental Section

### Materials

Niobium ethoxide ([Nb(C_2_H_5_O)_5_], Alfa Aesar, 99.9%), lithium acetate (CH_3_COOLi, Aladdin, 99.9%), lithium difluoro(oxalate)-borate (LiDFOB, Aladdin, 99%), LiNi_0.8_Co_0.1_Mn_0.1_O_2_ (NCM811, Hefei Kejing Material Technology Co. Ltd.), Li_6_PS_5_Cl (LPSCl, Hefei Kejing Material Technology Co. Ltd.), Ketjen Black (KB, Hefei Kejing Material Technology Co. Ltd.), Pt powder (Macklin, 99.9%), and Al (Alfa Aesar, 20 μm thickness) were purchased without further purification.

### Preparation of LiNbO_3_@NCM811, LiDFOB@LPSCl, Pre-Lithiated Al Anode, and Composite Cathode

#### ***Preparations of the LiNbO***_***3***_***@NCM811***

0.0194 g of niobium ethoxide and 0.004 g of lithium acetate (molar ratio 1:1) were dissolved in anhydrous ethanol and stirred for 12 h. Then 0.973 g NCM811 was added and ultrasonically dispersed for 2 h. After homogeneous dispersion by ultrasonication, the mixture was stirred for another 1 h and then transferred in a vacuum oven to remove the solvents and obtain the LiNbO_3_-coated NCM811 powder. The powder was annealed in air at 450 °C for 1 h to obtain the final product, denoted as LiNbO_3_@NCM811.

#### Preparations of the LiDFOB@LPSCl

LPSCl and LiDFOB were weighed according to the mass ratios of 100:0.5, 100:1, 100:1.5, and 100:2, respectively, and mixed in a mortar. The above operations were carried out in a glove box (H_2_O < 0.01 ppm, O_2_ < 0.01 ppm). The mixture was poured into a ball milling jar and milled under Ar atmosphere for 15 h at a speed of 500 r min^−1^ with a ball-to-feed ratio of 50:1. The obtained sample is denoted as LiDFOB@LPSCl.

#### Preparations of the Pre-Lithiated Al Anode

The Al foil with a diameter of 10 mm was cleaned with anhydrous ethanol before use. Li foil with an optimal molar ratio of Li:Al = 0.14:1 was pressed onto the Al foil, and then, 300 MPa pressure was applied and kept for 6 h. The above operation was conducted in a glove box.

#### Preparations of the Composite Cathode

The prepared LiNbO_3_@NCM811 and sulfide electrolytes (LiDFOB@LPSCl or bare LPSCl) were put into a mortar with a mass ratio of 7:3 and ground for 0.5 h to ensure homogeneous mixing. The above operation was conducted in a glove box.

### Cell Assembly

For the assembly of D-cathode/LiIn or M-cathode/LiIn battery, 120 mg LPSCl was added into a poly(ether-ether-ketone) (PEEK) mold with an internal diameter of 10 mm and 280 MPa pressure was applied and held for 2 min (The thickness of the molded electrolyte pellet is ~ 1.2 mm). Then the prepared composite cathode was evenly dispersed on the one side of the LPSCl pellet and 280 MPa pressure was applied and held for 5 min. In foil was placed on the other side of the LPSCl pellet and a Li foil with a molar ratio of Li:In = 0.5:1 was pressed on the In foil. Carbon-coated Al and Cu foils were used as the collectors at the cathode and anode sides, respectively. The assembled battery was operated under pressure of 80 MPa by a stainless steel frame. For the assembly of Li-Al/D-cathode battery, the procedures remain the same except that the prepared pre-lithiated Al anode was used to replace LiIn and no additional Cu foil was added as the collector. The symmetric cells were assembled with the same method except that the pre-lithiated Al or Li anode was placed on both sides of the LPSCl pellet. As for the assembly of the Li-Al/D-cathode pouch cell, firstly, the prepared composite cathode was thoroughly mixed with polytetrafluoroethylene with a mass ratio of 99:1 and ground with an agate mortar until a dough was formed. Then the dough was roll-pressed into a sheet with desired thickness to obtain the cathode film. The LPSCl film was fabricated following similar procedures. Finally, the cathode film, LPSCl film, and the freestanding pre-lithiated Al anode were pressed together and vacuum-sealed in aluminum plastic film. Nickel and aluminum metal tabs were welded to anode and cathode side as collectors, respectively.

LiDFOB@LPSCl-KB/LPSCl/LiIn battery: LiDFOB@LPSCl and KB mixture was obtained by grinding the prepared LiDFOB@LPSCl and KB with a mass ratio of 1:1 for 1 h. The LiDFOB@LPSCl-KB/LPSCl/LiIn battery was assembled with the same method as D-cathode/LiIn or M-cathode/LiIn battery except the change of the electrodes. The LiDFOB@LPSCl-KB (10 mg) mixture was adopted as the working electrode and distributed evenly on the one side of the LPSCl pellet. The LPSCl-KB/LPSCl/LiIn or LPSCl-Pt/LPSCl/LiIn battery was fabricated following similar procedures.

Al/LPSCl/LiIn battery: The Al/LPSCl/LiIn battery was assembled with the same method as LiDFOB@LPSCl-KB/LPSCl/LiIn battery except that the LiDFOB@LPSCl-KB mixture was changed to an Al foil with a diameter of 10 mm.

### Electrochemical Tests

The galvanostatic discharge/charge tests of the ASSLBs were carried out on the Neware battery test system. Cyclic voltammetry and linear sweep voltammetry tests were conducted on the CHI with a scan rate of 0.1 mV s^−1^. Electrochemical impedance spectroscopy (EIS) tests were performed on the Solartron with an applied frequency range of 0.1–10^6^ Hz and an amplitude of 5 mV. The voltage ranges were set to 2.1–3.78 V versus LiIn for D-cathode/LiIn or M-cathode/LiIn battery and 2.4–4.07 V versus Li-Al for Li-Al/D-cathode battery. Specific capacity calculations were based on the mass of LiNbO_3_@NCM811. The N/P ratio is defined as the ratio between the maximum capacity of the pre-lithiated Al anode under conditions of maintaining a constant potential (3 mAh) and the theoretical capacity that can be provided by the composite cathode. For the Li-Al/D-cathode battery with a N/P ratio of 1.1, the mass of the pre-lithiated Al anode and composite cathode was 4.3 and 20 mg, respectively.

### Material Characterization

X-ray diffraction (XRD, Bruker D8) analysis was carried out to analyze the structure change of NCM811 and LPSCl after modification. The morphology observation was conducted by scanning electron microscopy (SEM, Hitachi SU8010). The successful formation of the coating layer on NCM811 and LPSCl was demonstrated by transmission electron microscopy (TEM, FEI TF20) and cryo-TEM, respectively. And the corresponding composition mapping was obtained by the equipped energy-dispersive X-ray spectrometry (EDS). X-ray photoelectron spectroscopy (XPS) test was performed on PHI 5000 VersaProbe-II to analyze the specific components of the coating layer on LPSCl and structure change of LPSCl after cycling. The cross-sectional microstructure of the composite cathode before and after cycling was observed by focused ion/electron dual-beam electron microscopy (Helios G4 CX). Atomic force microscopy (AFM, Bruker Dimension ICON) and RTESP-525 tip were used to analyze the Young’s modulus of the LiDFOB@LPSCl and LPSCl electrolytes. Raman spectroscopy was carried out on a confocal Raman microscope (inVia, Renishaw) with an excitation wavelength of 633 nm. Time-of-flight secondary-ion mass spectrometry (ToF–SIMS) measurements were taken via a TOF-SIMS5 (ION-TOF-GmbH) in Nano-X. And a pulsed Bi^3+^ ion beam (30 keV) set in high current mode was applied for ToF–SIMS surface analysis.

## Results and Discussion

### Practical Stability Window of LPSCl and Electrochemical Property of Al

The stability of the electrode–electrolyte interface is intimately linked to the electrochemical stability window (ESW) of the electrolyte. Theoretical calculations indicate that the ESW of LPSCl ranges from 1.71 to 2.01 V versus Li/Li^+^, suggesting that LPSCl undergoes degradation when the applied voltage falls below 1.71 V or exceeds 2.01 V [14]. Despite this, anodes such as Li-In alloy and Li_4_Ti_5_O_12_, which operate at potentials below 1.71 V, have been identified as stable in LPSCl-based batteries, implying that the practical stability window for LPSCl may indeed be broader than the theoretical predictions.

**Fig. 2:**
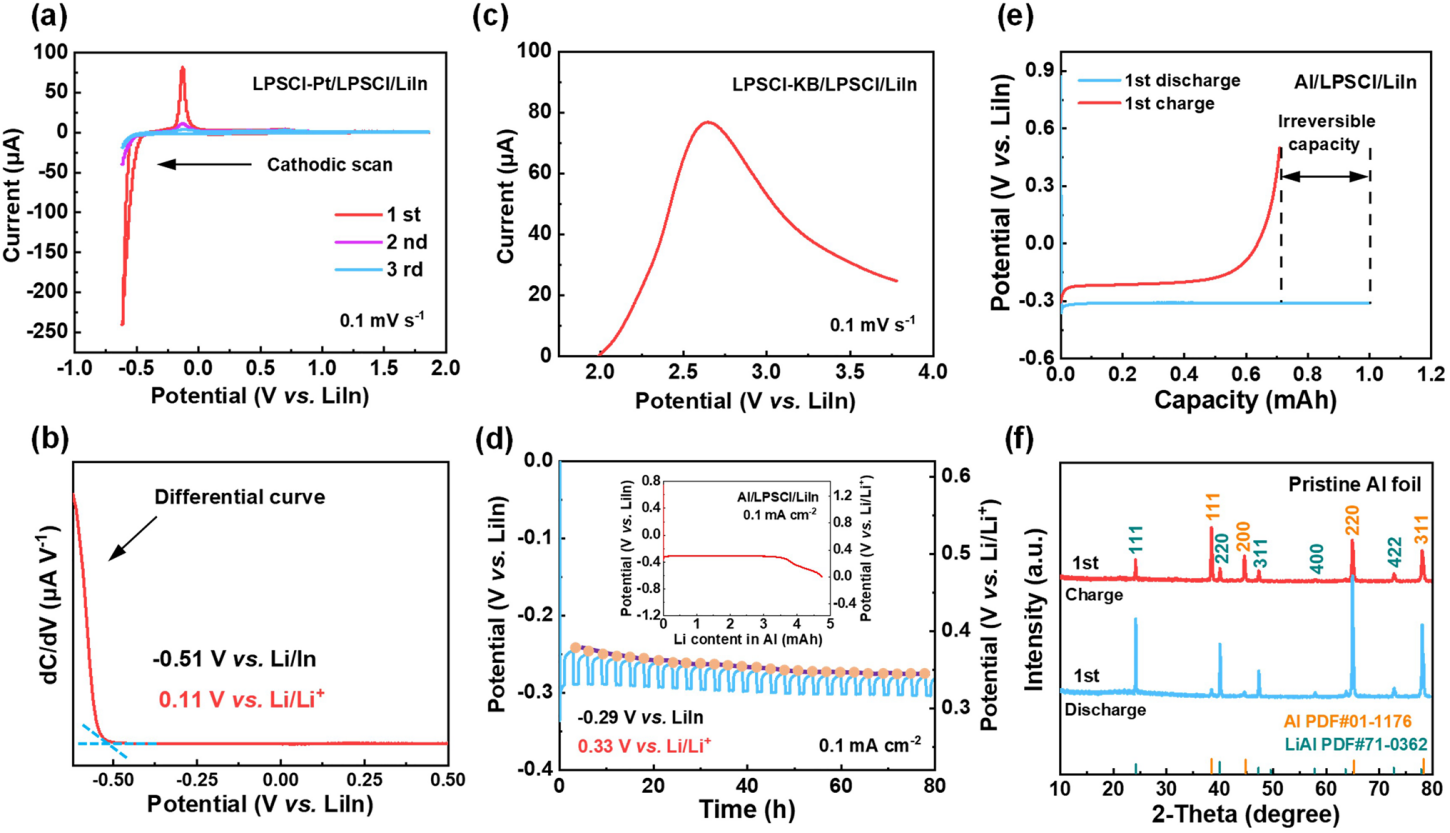
Practical ESW of the LPSCl electrolyte and the lithiation behaviors of Al. **a** CV test of the LPSCl-Pt/LPSCl/LiIn battery at a scan rate of 0.1 mV s^−1^. **b** Differential curve of the first cathodic scan in **a**. **c** Linear sweep voltammetry test of the LPSCl-KB/LPSCl/LiIn battery at a scan rate of 0.1 mV s^−1^. **d** GITT profile of the Al foil. The current pulse was 0.1 mA cm^−2^ and for 1 h, followed by a rest time for 2 h. Inset is the lithiation profile of Al in the Al/LPSCl/LiIn battery at a current density of 0.1 mA cm^–2^. **e** Initial discharge and charge profiles of the Al/LPSCl/LiIn battery. The discharge capacity was set at 1 mAh, and the charging cutoff voltage was set at 0.5 V vs. LiIn. **f** XRD pattern of the pristine Al foil after initial discharge (blue) and charge (red)

To offer practical guidance for LPSCl applications, the ESW of LPSCl under operational conditions was investigated. The practical reductive stability limit of LPSCl was determined via cyclic voltammetry (CV) experiments. The experimental setup featured a LPSCl-Pt/LPSCl/LiIn configuration, where platinum (Pt) powder was introduced to the working electrode in lieu of carbon materials to enhance electrical contact area and prevent carbon lithiation at low potentials. The Li-In alloy, with a molar ratio of Li:In at 0.5:1 and a stable potential of 0.62 V versus Li/Li^+^, demonstrated exceptional compatibility with LPSCl, thus serving as both counter and reference electrodes in the study [13]. As depicted in Fig. 2a, a discernible reduction current peak emerged at low potentials during the initial cathodic sweep, indicating the decomposition of LPSCl. The corresponding differential curve in Fig. 2b reveals a sharp increase at − 0.51 V versus LiIn (0.11 V vs. Li/Li^+^), which suggests that the reductive stability limit of LPSCl is 0.11 V versus Li/Li^+^. The oxidation peak exhibits reduced intensity during the subsequent anodic scan, implying that the electrochemical reduction of LPSCl is not entirely reversible. The second and third cycles display diminished reduction and oxidation current peaks, likely due to the passivation effect of the decomposition products. The oxidative behavior of LPSCl was examined using a linear sweep voltammetry test. As illustrated in Fig. 2c, the decomposition current of the LPSCl electrolyte was observable from the open-circuit voltage (2.5 V vs. Li/Li^+^) of the battery up to the cutoff voltage of 3.78 V versus LiIn (4.4 V vs. Li/Li^+^), suggesting that the actual oxidation limit of LPSCl is less than 2.5 V versus Li/Li^+^.

The characteristics of Al foil were studied to estimate its feasibility as the anode of ASSLBs with LPSCl electrolyte. The equilibrium potential of Al after lithiation was measured by the galvanostatic intermittent titration technique (GITT) test. As shown in Fig. 2d, the lithiated Al foil presented a stable potential of − 0.29 V versus LiIn (0.33 V vs. Li/Li^+^). The moderate operating potential of Al foil is higher than the decomposition potential of LPSCl, guaranteeing the anode–electrolyte interface stability. Quantitative lithiation test of Al foil was also carried out to reveal the maximum Li content that can be accommodated in Al (inset in Fig. 2d). The Al foil maintains a constant potential until the Li content in Al exceeds 3.6 mAh (4.6 mAh cm^−2^), which demonstrates that the Al foil can enable a commercially relevant capacity (2–5 mAh cm^−2^) while still retaining interface stability with the LPSCl electrolyte. The electrochemical performance of Al foil was investigated. Figure 2e displays the initial discharge–charge profiles of the Al/LPSCl/LiIn battery with a Coulombic efficiency of 70.9%, which indicates that a portion of Li ions cannot be reversibly released after entering into the Al skeleton, as further evidenced by the X-ray diffraction (XRD) results presented in Fig. 2f. In the subsequent cycles, the irreversible capacity in Al foil gradually increased until the Coulombic efficiency of the battery reached ~ 100% after 25 cycles (Fig. S1).

### Interface Stability Between the Pre-Lithiated Al Anode and LPSCl

To improve the reversibility of the Al foil, an anode pre-lithiation technique was employed. In this study, the optimal amount of implanted Li in the Al matrix was determined to be 0.6 mAh (corresponding to a Li:Al molar ratio of 0.14:1). This quantity effectively compensates for irreversible capacity losses in the Al matrix while preserving sufficient sites for Li^+^ released from the cathode (Fig. S2). Pre-lithiation of the Al foil was achieved via a mechanical alloying method. As illustrated in Fig. S3, the continuous pressure applied during this process accelerates the diffusion of Li atoms into Al matrix at the interface, promoting a solid-state reaction to form the Li-Al alloy [13]. XRD analysis confirmed that the pre-lithiated Al foil consists of a mixture of Al and Li-Al phases (Fig. S4), which indicates that the lithiation of Al proceeds through a biphasic reaction characterized by a constant potential, consistent with the flat potential curve shown in Fig. 2d. SEM was utilized to characterize the pre-lithiated Al foil. As shown in Fig. S5, the surface and cross-sectional morphologies exhibit a dense and uniform structure.

**Fig. 3:**
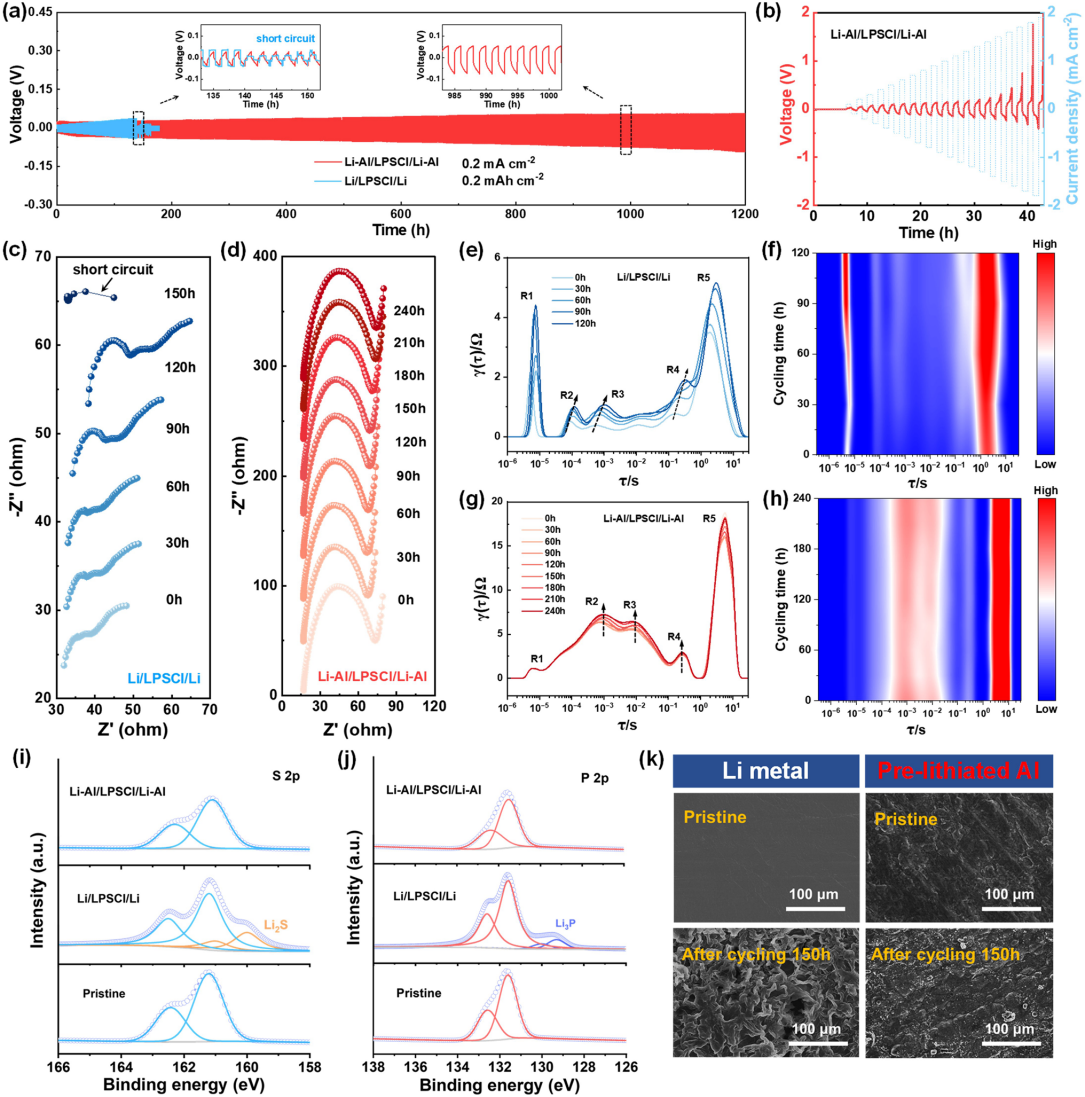
Compatibility evaluation between the pre-lithiated Al anode and LPSCl electrolyte. **a** Galvanostatic Li plating/stripping profiles of the Li/LPSCl/Li (blue) and Li-Al/LPSCl/Li-Al (red) cells at 0.2 mA cm^−2^ and 0.2 mAh cm^−2^. (Inset is the enlarged galvanostatic Li plating/stripping profiles.) **b** Critical current density of the Li-Al/LPSCl/Li-Al cell. Nyquist plots of **c** Li/LPSCl/Li cell and **d** Li-Al/LPSCl/Li-Al cell after different cycling time. **e–h** DRT analyses of Li/LPSCl/Li and Li-Al/LPSCl/Li-Al cells after different cycling time. **i** S 2*p* and **j** P 2*p* X-ray photoelectron spectroscopy of the LPSCl electrolyte. **k** Scanning electron microscopy images of the Li and pre-lithiated Al anodes before and after cycling 150 h

Symmetric Li-Al/LPSCl/Li-Al cell (note: Li-Al refers to the pre-lithiated Al foil) was fabricated to evaluate the interfacial stability between the pre-lithiated Al anode and LPSCl electrolyte. As shown in Fig. 3a, the Li-Al/LPSCl/Li-Al cell exhibits excellent cycling stability with a small polarization at 0.2 mA cm^−2^ (0.2 mAh cm^−2^) after 1200 h. For comparison, a symmetric Li/LPSCl/Li cell was assembled and tested under the same conditions, which suffered from a short circuit after cycling for 140 h, indicating that Li dendrites had pierced through the electrolyte. The Li-Al/LPSCl/Li-Al cell was evaluated under deeper charge–discharge conditions (0.5 mA cm^−2^, 0.5 mAh cm^−2^) and demonstrated stable cycling for over 1000 h, as shown in Fig. S6. Critical current density (CCD) tests were also conducted to determine the maximum current density that the Li-Al/LPSCl/Li-Al cell can tolerate without failure. As depicted in Fig. 3b, although the overpotential increased gradually with the rise in current density, no signs of cell failure were observed. In contrast, short circuits occurred in the Li/LPSCl/Li cell when the current density reached 0.9 mA cm^−2^ (Fig. S7).

EIS measurements were taken to probe into the internal impedance variation of the Li/LPSCl/Li and Li-Al/LPSCl/Li-Al cells. The Li/LPSCl/Li experienced cell failure before 150 h (Fig. 3c). Conversely, the Li-Al/LPSCl/Li-Al cell maintained a stable impedance profile, with no indication of short-circuiting within the 240 h timeframe (Fig. 3d). To gain deeper insights into the impedance characteristics, the EIS data were further deconvoluted using semiquantitative distribution of relaxation times (DRT) analysis. As depicted in Fig. 3e-h, five distinct relaxation processes (R1–R5) were identified [15]. R1 corresponds to the bulk electrolyte resistance, while R2 and R3 reflect the SEI impedance. R4 is attributed to charge transfer resistance, and R5 represents diffusion impedance within the electrodes. Notably, a low time constant (τ) in the R2 and R3 regions indicates rapid Li-ion transport kinetics across the interphase, while a low τ value in the R4 region implies an enhanced response for charge transfer. Due to the unstable interface between Li and LPSCl, both the SEI resistance (*R*_SEI_) and charge transfer resistance (*R*_ct_) progressively increase in the Li/LPSCl/Li cell over extended cycling. In stark contrast, the *R*_SEI_ and *R*_ct_ of Li-Al/LPSCl/Li-Al cell could remain stable with slight changes.

XPS was used to study the composition change of the LPSCl electrolyte after the Li/LPSCl/Li and Li-Al/LPSCl/Li-Al cell cycling for 150 h (Fig. 3i, j). The XPS S 2*p* and P 2*p* spectra collected at the Li-Al/LPSCl interface maintain consistency with the characteristic peaks of the pristine LPSCl, while new peaks representing the reduced products of Li_2_S and Li_3_P can be detected at the Li/LPSCl interface. The above discoveries demonstrate that the pre-lithiated Al anode has better interfacial compatibility with LPSCl compared to Li anode. In addition, the postmortem characterizations were also conducted by SEM (Fig. 3k). Compared with the pristine state, neither dendrites nor pulverization is observed on the pre-lithiated Al anode, while obvious Li dendrites appeared on Li anode after cycling 150 h.

### Interfacial Stability Between NCM811 and LPSCl

The electrochemical window of LPSCl is limited, rendering it prone to interfacial incompatibility when paired with high-voltage oxide cathodes that operate above 4.2 V versus Li/Li⁺ [16]. To mitigate this issue, coating CAMs with a stabilizing layer has emerged as a viable strategy to enhance interfacial stability with the electrolyte [17]. However, technical hurdles and the imperative to ensure sufficient electronic conductivity within the active material during charge and discharge often result in incomplete surface coatings on CAM particles. Consequently, interfacial side reactions can still be triggered between these partially coated CAM particles and the electrolyte.

To overcome the interfacial incompatibility between high-nickel CAMs and LPSCl electrolyte, we employed a dual-reinforcement approach, wherein concurrent modifications to both the CAMs and electrolyte were implemented to synergistically enhance their interfacial stability. Lithium niobate (LiNbO_3_), known for its good ionic conductivity and electrical insulating properties [17], was applied as a protective coating on NCM811. Characterization (Fig. S8) confirmed the successful synthesis of an amorphous LiNbO_3_ layer, approximately 3.2 nm thick, on the NCM811 surface (denoted as LiNbO_3_@NCM811). Furthermore, to improve the oxidative stability of LPSCl at high potentials and to mitigate side reactions between any uncoated areas of NCM811 particles and the LPSCl electrolyte, lithium difluoro(oxalate)borate (LiDFOB) was incorporated as a functional additive into the LPSCl electrolyte. The synthesis procedure is illustrated in Fig. S9, and the resulting electrolyte is referred to as LiDFOB@LPSCl.

To ascertain the optimal concentration of the LiDFOB additive, we investigated the ionic conductivity of LiDFOB@LPSCl electrolytes and evaluated the cycling stability of batteries assembled with varying amounts of LiDFOB. As depicted in Fig. S10a, b, the ionic conductivity of LiDFOB@LPSCl demonstrated a consistent decline with increasing LiDFOB content. Concerning the cycling stability of the batteries (Fig. S10c, d), the most favorable electrochemical performance was observed when the mass fraction of LiDFOB reached 1 wt%. This phenomenon can be attributed to the fact that an insufficient quantity of LiDFOB may not adequately form a protective layer on the LPSCl surface, whereas an excessive amount of LiDFOB can significantly diminish Li⁺ conductivity, resulting in pronounced polarization effects.

**Fig. 4:**
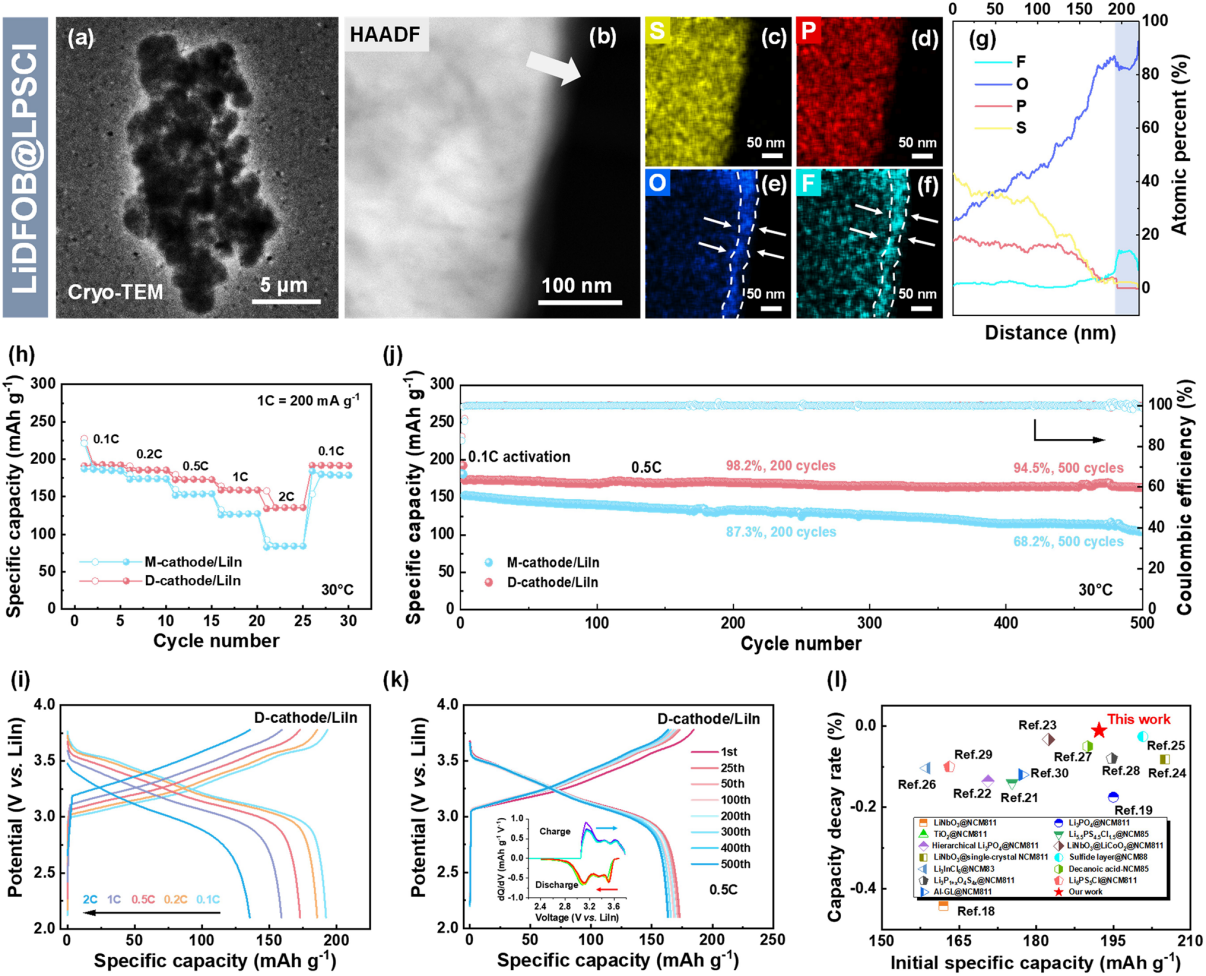
Characterization and electrochemical performance of the dual-reinforced cathode. **a** Cryo-TEM image of LiDFOB@LPSCl. **b** HAADF image and **c-f** corresponding elemental mapping images of LiDFOB@LPSCl. **g** Linear scans in the direction of the arrow in **b**. **h** Rate performance and **i** charge/discharge curves of the D-cathode/LiIn battery at various rates ranging from 0.1C to 2.0C. **j** Long-term cycling performance of the D-cathode/LiIn battery and **k** charge/discharge curves at different cycles (inset: corresponding dQ/dV curves). **l** Comparison of initial specific capacity and capacity decay rate per cycle between previous work and this study

Cryo-transmission electron microscopy (cryo-TEM) was used to investigate the LiDFOB@LPSCl electrolyte (Fig. 4a). The corresponding EDS mappings (Fig. 4b–f) and linear scans (Fig. 4g) reveal an additional layer, 15–25 nm thick, on the LPSCl surface. XPS tests were conducted to determine the specific composition of the coating layer. As shown in Fig. S11, the results indicate that the surface layer of LPSCl consists of inorganic (LiF, Li_*x*_PO_*y*_F_*z*_, and LiBO_2_) and organic (B-C) components. CV measurements were taken on LiDFOB@LPSCl to demonstrate that LiDFOB addition enhances the stability of LPSCl under high-potential conditions. No significant peaks were observed during cathodic and anodic scans (Fig. S12), indicating that the oxidation tolerance of LPSCl was effectively promoted.

To validate the enhanced stability of nickel-rich cathodes via concurrent modification of NCM811 and LPSCl, we fabricated an ASSLB comprising a dual-reinforced cathode, wherein LiNbO_3_@NCM811 served as the active material and LiDFOB@LPSCl acted as the electrolyte, paired with a LiIn anode (denoted as D-cathode/LiIn). For comparative purposes, a reference ASSLB was also assembled, featuring a mono-modified cathode with LiNbO_3_@NCM811 as the active material and pristine LPSCl as the electrolyte, paired with a LiIn anode (denoted as M-cathode/LiIn).

The electrochemical performances of the D-cathode/LiIn and M-cathode/LiIn batteries were evaluated at various current densities. As displayed in Fig. 4h, the D-cathode/LiIn battery delivered reversible capacities of 191.2, 185.3, 172.5, 158.8, and 134.3 mAh g^−1^ at 0.1C, 0.2C, 0.5C, 1C, and 2C, respectively. The capacity recovered to 191.8 mAh g^−1^ when the current density was restored to 0.1C. Under the same conditions, the M-cathode/LiIn battery delivered lower reversible capacities of 187.0, 173.2, 151.7, 125.9, and 82.8 mAh g^−1^. The corresponding charge/discharge curves at different rates are shown in Figs. 4i and S13. The D-cathode/LiIn battery exhibited better reversibility and lower voltage polarization than the M-cathode/LiIn, indicating improved interfacial compatibility between NCM811 and LPSCl.

The cycling properties of the D-cathode/LiIn and M-cathode/LiIn batteries are illustrated in Fig. 4j. The D-cathode/LiIn battery delivered an initial reversible specific capacity of 192.2 mAh g^−1^ with a decent initial coulombic efficiency (ICE) of 84.8%. At a higher current density of 0.5C, the battery achieved a reversible specific capacity of 172.6 mAh g^−1^ and operated steadily for over 500 cycles with an excellent capacity retention of 94.5%. In comparison, the M-cathode/LiIn battery exhibited a lower ICE of 82.8% and a capacity retention of 68.2% after 500 cycles. The charge/discharge curves of the D-cathode/LiIn and M-cathode/LiIn batteries at different cycles are displayed in Figs. 4k and S14. The well-overlapped curves of the D-cathode/LiIn battery imply excellent cycling stability. Additionally, the differential capacity (dQ/dV) analysis of the D-cathode/LiIn battery (inset in Fig. 4k) shows that the intensity of the dQ/dV peaks did not decrease or shift significantly even after 500 cycles, which indicates that the internal structure of the electrode was well preserved. The electrochemical performance of the D-cathode was carefully compared with other similar cathodes reported previously (Fig. 4l), demonstrating its great competitiveness in terms of reversible capacity and cycling stability [18–30].

**Fig. 5:**
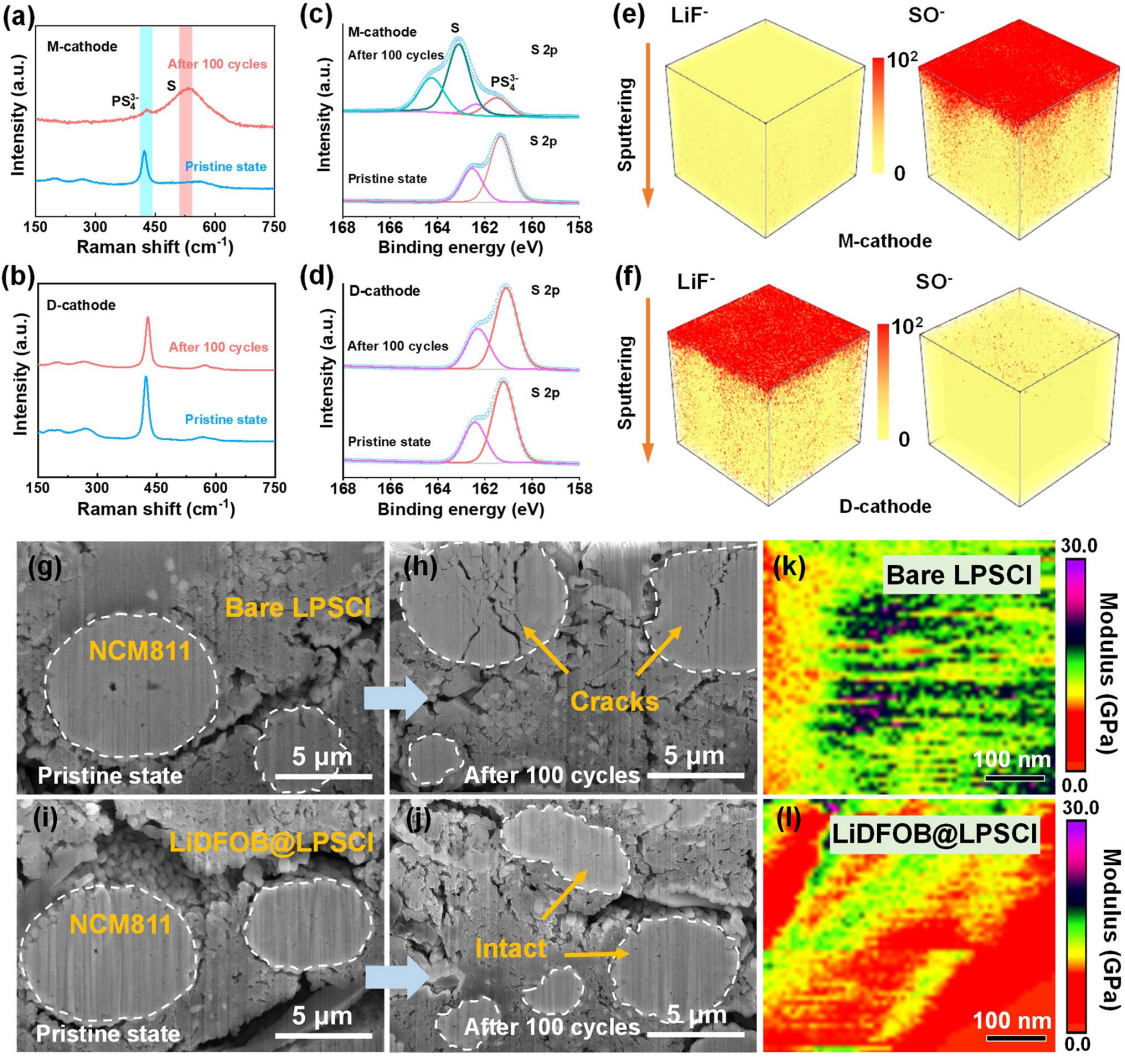
Composition and structure investigation of the M-cathode and D-cathode. **a, b** Raman and **c, d** XPS results of the M-cathode and D-cathode before and after cycling for 100 cycles. ToF–SIMS 3D reconstructed images of **e** the M-cathode and **f** D-cathode. Cross-sectional FIB-SEM images of **g, h** M-cathode and **i, j** D-cathode before and after cycling for 100 cycles. Areal distribution of Young’s modulus of **k** bare LPSCl and **l** LiDFOB@LPSCl electrolytes

To elucidate the mechanisms underlying the improved battery performance achieved through the dual-reinforcement strategy, the impedance evolution of the M-cathode/LiIn and D-cathode/LiIn batteries was analyzed. As shown in Fig. S15a, the initial interfacial impedances of the M-cathode/LiIn and D-cathode/LiIn batteries were 25.9 and 54.5 Ω, respectively. The higher initial impedance of the D-cathode/LiIn cell can be attributed to the additional protective layer on LPSCl. However, the interfacial impedance of the M-cathode/LiIn battery increased substantially to 358.7 Ω after 100 cycles, whereas the D-cathode/LiIn battery exhibited a considerably lower impedance of 129.9 Ω (Fig. S15b). To further investigate the compositional changes within the cathodes, Raman spectroscopy and XPS analyses were performed. As depicted in Fig. 5a, c, a distinct oxidation product of sulfur (S) was detected in the M-cathode after 100 cycles, indicating severe decomposition of LPSCl. In contrast, the Raman and XPS spectra of the D-cathode remained nearly unchanged (Fig. 5b, d).

Furthermore, time-of-flight secondary-ion mass spectrometry (ToF–SIMS) was employed to investigate the surface-to-bulk composition distribution in the cycled cathodes. As shown in Fig. S16, with increasing sputtering time, the content of the LiF^−^ and SO^−^ ionic fragments (characteristic of LiF and S, respectively) decreased and reached equilibrium after 800 s of sputtering in both M-cathode and D-cathode. The corresponding three-dimensional reconstructed spatial distributions of each component are presented in Fig. 5e, f, where the intensity of the red color represents the concentration of the ionic fragments. In the M-cathode, the LiF^−^ signal was nearly absent, while a high concentration of the SO^−^ component was observed, pointing to severe decomposition of the LPSCl electrolyte [31]. In contrast, the D-cathode exhibited a detectable LiF^−^ signal from the surface layer of LPSCl, and the signal intensity of the SO^−^ fragment was weak, which demonstrates that the stability of LPSCl was effectively improved after modification.

Cross-sectional focused ion beam (FIB)-SEM characterizations were conducted to visualize the structural variations in the cathodes of the M-cathode/LiIn and D-cathode/LiIn batteries. As shown in Fig. 5g-j, obvious cracks appeared in NCM811 particles of the M-cathode after 100 cycles, which suggests that severe stress concentrations occurred in the M-cathode after cycling. In stark contrast, the cycled D-cathode remained superior structural integrity without cracks or pulverizations. Atomic force microscopy tests were performed on the LPSCl and LiDFOB@LPSCl electrolytes to understand their surface properties. As displayed in Figs. 5k, l, and S17, the bare LPSCl, ball-milled LPSCl and LiDFOB@LPSCl exhibited average Young’s modulus of 15.7, 17, and 9.7 GPa, respectively. The lower Young’s modulus of LiDFOB@LPSCl than those of the bare LPSCl and ball-milled LPSCl electrolytes could be attributed to the good flexibility of the C-B organic component formed on LPSCl, which provided better buffering functions and alleviated the stresses generated inside the electrodes.

### Electrochemical Performances of the All-Solid-State Li-Al/D-Cathode Battery

**Fig. 6:**
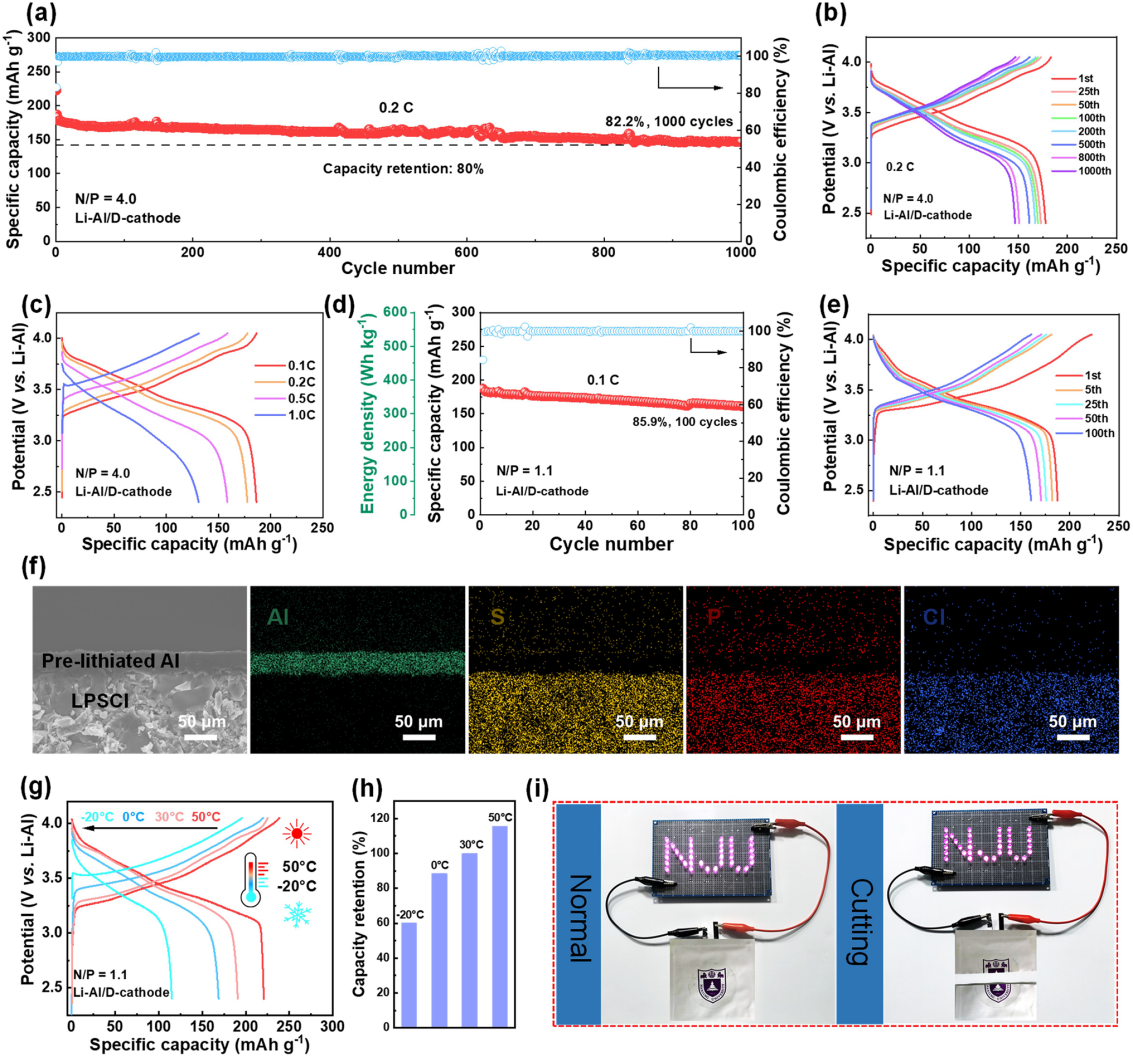
Electrochemical tests of the designed all-solid-state Li-Al/D-cathode battery. **a** Long-term cycling performance at 0.2C with a N/P ratio of 4.0 (activation at 0.1C for the first two cycles) and **b** corresponding charge–discharge curves at different cycles. **c** Charge/discharge curves at various rates ranging from 0.1C to 1.0 C. **d** Cycling performance at 0.1C with a N/P ratio of 1.1 and **e** corresponding charge–discharge curves at different cycles. **f** Interfacial morphology and corresponding EDS mappings between the pre-lithiated Al anode and LPSCl electrolyte after cycling for 100 cycles. **g** Charge–discharge curves and **h** corresponding capacity retention at different temperature ranging from − 20 °C to 50 °C. **i** Illustration of the Li-Al/D-cathode pouch cell powering an LED light

After achieving excellent reversibility of the negative and positive electrodes and superior interfacial compatibility with the sulfide electrolyte, a promising type of ASSLB with different battery configuration was further fabricated, comprising the pre-lithiated Al anode, dual-reinforced NCM811 cathode, and LPSCl electrolyte, denoted as Li-Al/D-cathode. The long-term cycling stability of the Li-Al/D-cathode battery was evaluated. As shown in Fig. 6a, b, the Li-Al/D-cathode battery with a N/P ratio of 4.0 presented an initial reversible capacity of 185.0 mAh g^−1^ at 0.1C and operated steadily for more than 1000 cycles at 0.2C with an excellent capacity retention of 82.2%. Rate performance of the Li-Al/D-cathode battery was also explored and displayed in Figs. 6c and S18. At current densities of 0.1C, 0.2C, 0.5C and 1C, the Li-Al/D-cathode battery delivered reversible capacities of 185.0, 177.7, 160.0, and 131.7 mAh g^−1^, respectively.

The performance of the Li-Al/D-cathode battery was evaluated under stringent conditions to assess its durability for practical applications. Given that cathode areal capacity is a key factor influencing overall battery energy density, the N/P ratio of the Li-Al/D-cathode battery was reduced to 2.0 by increasing the cathode loading. As shown in Fig. S19, the battery delivered an initial reversible specific capacity of 182.2 mAh g^−1^ at 0.1C, with an impressive capacity retention of 88.1% after 200 cycles. Furthermore, the Li-Al/D-cathode battery with a critical N/P ratio of 1.1 was developed, increasing the areal capacity of the NCM811 cathode to 3.5 mAh cm^−2^. The battery exhibited a reversible capacity of 187.5 mAh g^−1^ at 0.1C, corresponding to an energy density of 375 Wh kg^−1^ (calculated based on the total mass of the positive and negative electrodes). Even after more than 100 cycles, it maintained a high energy density of 322 Wh kg^−1^ with a capacity retention of 85.9% (Fig. 6d, e). Post-cycling SEM analysis of the anode–electrolyte cross section revealed that the pre-lithiated Al anode maintained intimate contact with the LPSCl electrolyte after 100 cycles, demonstrating excellent interfacial stability (Fig. 6f).

The electrochemical performance of the Li-Al/D-cathode battery (N/P = 1.1) was also evaluated across a wide temperature range. As shown in Fig. S20, the battery delivered reversible specific capacities of 220.6, 190.7, 168.8, and 114.9 mAh g^−1^ at 50, 30, 0, and − 20 °C, respectively. Notably, when the temperature was restored to 50 °C, the capacity recovered to 189.3 mAh g^−1^. The corresponding charge/discharge profiles of the Li-Al/D-cathode battery at different temperatures are presented in Fig. 6g. Even at − 20 °C, the battery retained 60.3% of its reversible capacity measured at 30 °C, despite increased polarization at lower temperatures (Fig. 6h), demonstrating its excellent reversibility. Additionally, an Li-Al/D-cathode pouch cell was fabricated. As depicted in Fig. 6i, the battery successfully powered an LED light with the “NJU” pattern, and the LED light remained fully operational even when the battery was cut open in the air.

## Conclusions

In summary, a promising type of ASSLB with high specific energy and long cycle stability was successfully constructed using high-capacity pre-lithiated Al anode, high-voltage NCM811 cathode, and LPSCl electrolyte. To provide guidance for the practical application of LPSCl, we firstly explored the ESW of LPSCl under operating conditions and determined the reduction (0.11 V vs. Li/Li^+^) and oxidation (< 2.5 V vs. Li/Li^+^) limit of LPSCl, respectively. Then, we demonstrated that Al can enable a commercial capacity of 4.6 mAh cm^−2^ while retaining a stable working potential of 0.33 V versus Li/Li^+^. To address the issues of poor reversibility of Al, anode pre-lithiation technique was adopted. The fabricated pre-lithiated Al anode shows superior interface stability with LPSCl, as verified by the steady operation of the Li-Al/LPSCl/Li-Al symmetric cell for over 1200 h at 0.2 mA cm^−2^. In addition, to resolve the interfacial instability between the CAMs and LPSCl, a dual-reinforcement technology was employed, where NCM811 and LPSCl particles in the composite cathode were simultaneously protected with LiNbO_3_ and LiDFOB, respectively. The assembled ASSLB composed of the dual-reinforced NCM811 cathode and LiIn anode (half-cell configuration) exhibits a high reversible capacity of 192.2 mAh g^−1^ with improved ICE of 84.8% at 0.1C, and achieved an excellent capacity retention of 94.5% at 0.5C after 500 cycles. Furthermore, the ASSLB composed of the pre-lithiated Al anode and dual-reinforced NCM811 cathode (full-cell configuration) was fabricated, which could operate steadily for more than 1000 cycles at 0.2C with a superb capacity retention of 82.2%. At a critical N/P ratio of 1.1, the battery achieved a reversible energy density of 375 Wh kg^−1^ with a retention of 85.9% after 100 cycles. Over a wide temperature range from − 20 to 50 °C, good reversibility and stability of the battery could still be attained. This work provides a promising anode selection for developing reliable ASSLBs with high energy and stability. Meanwhile, unlike many reports in the literature where two different electrolytes are required to guarantee cathode–electrolyte and anode–electrolyte interface stability, this work addressed the instability issues between the electrode materials and sulfide electrolyte, and realized stable electrochemical performances of all-solid-state battery by using one single electrolyte. In addition, considering the moderate potential, abundant reserves, and easy processability of Al, we also believe that the pre-lithiated Al anode combined with high-nickel cathode has great potential to realize an ASSLB with high safety and low cost, thus contributing to opening a new avenue for the practical ASSLBs.

## Supplementary Information

Below is the link to the electronic supplementary material.

Supplementary file1 (DOCX 6216 KB)
