# Supplementary material for: Developing High-Energy, Stable All-Solid-State Lithium Batteries Using Aluminum-Based Anodes and High-Nickel Cathodes
Source: Nanomicro Lett. 2025 Apr 29;17:239. doi: 10.1007/s40820-025-01751-y (PMC12040785; doi:10.1007/s40820-025-01751-y)
Supplement: Supplementary file 1 — Supplementary file1 (DOCX 6216 KB) [file 40820_2025_1751_MOESM1_ESM.docx]

Supporting Information for

**Developing High-Energy, Stable All-Solid-State Lithium Batteries Using Aluminum-Based Anodes and High-Nickel Cathodes**

Xin Wu^1†^, Meiyu Wang^2†^, Hui Pan^1^, Xinyi Sun^1^, Shaochun Tang^2*^, Haoshen Zhou^1^, and Ping He^1*^

^1^ Center of Energy Storage Materials & Technology, Department of Energy Science and Engineering, College of Engineering and Applied Sciences, Jiangsu Key Laboratory of Artificial Functional Materials, National Laboratory of Solid-State Microstructures, and Collaborative Innovation Center of Advanced Microstructures, Nanjing University, Nanjing 210093, P. R. China

^2^ Department of Materials Science and Engineering, College of Engineering and Applied Sciences, Jiangsu Key Laboratory of Artificial Functional Materials, National Laboratory of Solid-State Microstructures, and Collaborative Innovation Center of Advanced Microstructures, Nanjing University, Nanjing 210093, P. R. China

^†^Xin Wu and Meiyu Wang contributed equally to this work.

*Corresponding authors. E-mail: [pinghe@nju.edu.cn](mailto:pinghe@nju.edu.cn) (Ping He); [tangsc@nju.edu.cn](mailto:tangsc@nju.edu.cn) (Shaochun Tang)

**Supplementary Figures and Tables**


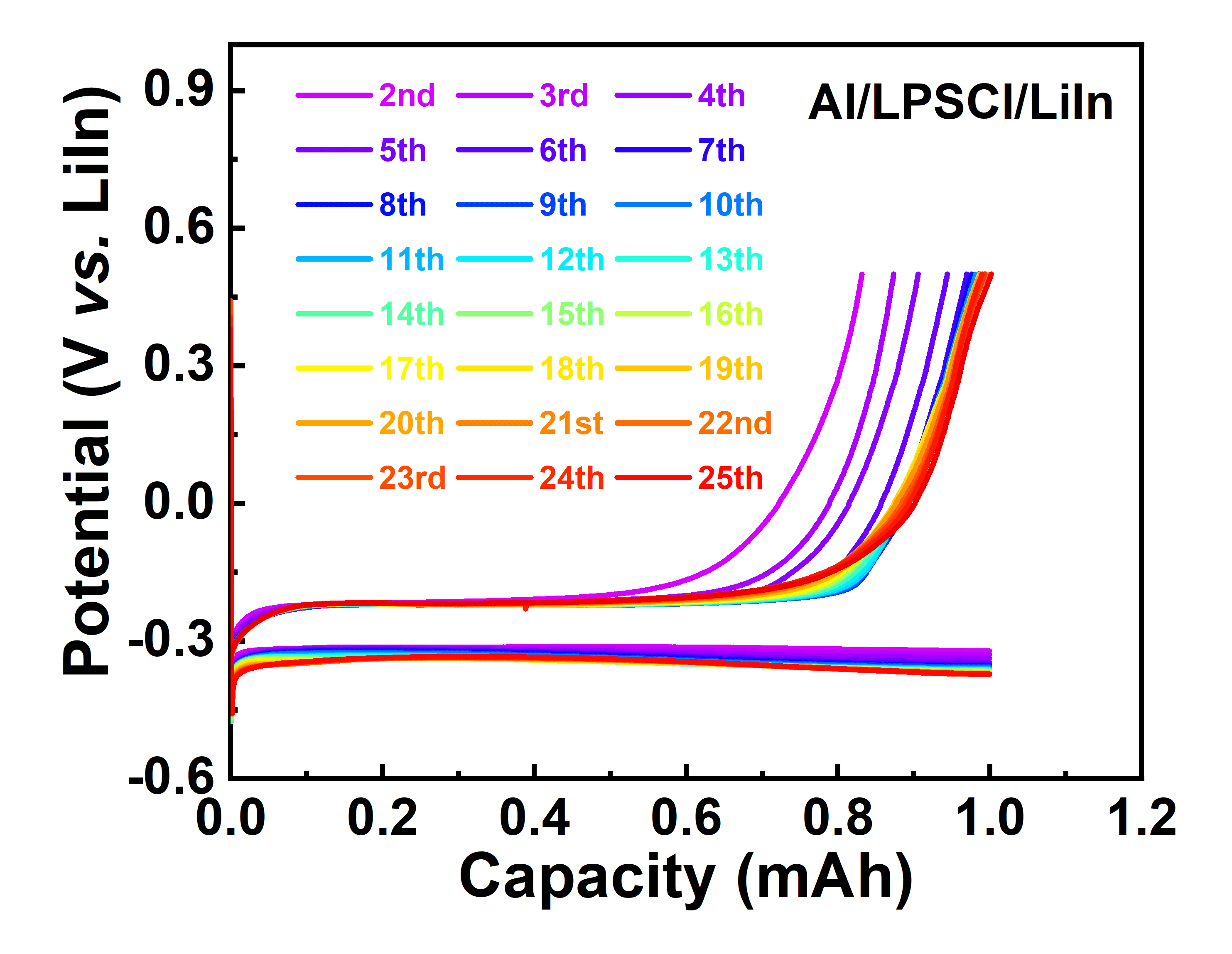


**Fig. S1** Discharge and charge profiles of the Al/LPSCl/LiIn battery. The discharge capacity was set at 1 mAh and the charging cutoff voltage was set at 0.5 V vs. LiIn. The irreversible capacity was determined to be 0.6 mAh.


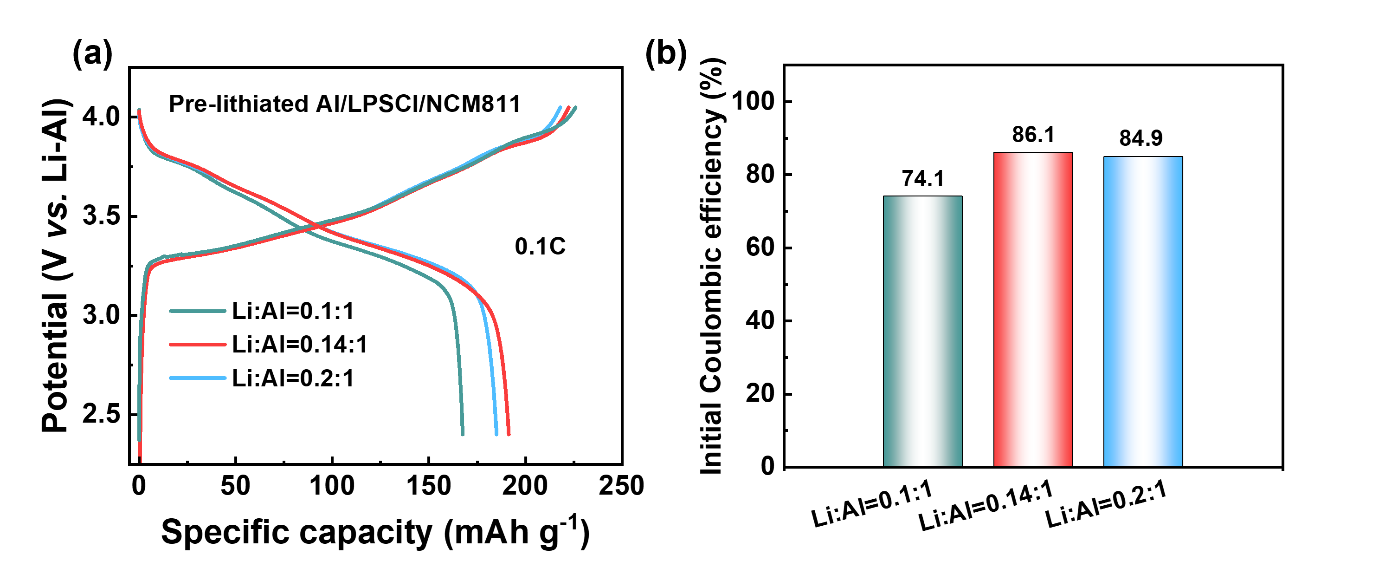


**Fig. S2** **a)** Charge/discharge profiles and **b)** initial Coulombic efficiency of the pre-lithiated Al/LPSCl/NCM811 battery with different molar ratio of Li to Al

According to the results of Fig. 2e and S1, the irreversible capacity in Al skeleton was determined to be 0.6 mAh, which indicates that pre-embedding 0.6 mAh of lithium into the Al matrix is necessary to ensure its excellent reversibility during cycling. Given that the theoretical capacity of the Al foil used in this work is 4.3 mAh, the corresponding Li:Al molar ratio was calculated to be 0.14:1.

If the pre-embedded lithium content is further increased (i.e., the Li:Al molar ratio exceeds 0.14), the number of available Li⁺ storage sites in the Al matrix decreases. This reduction limits the accommodation of Li⁺ extracted from the cathode during the initial charge, thereby lowering the battery’s first-cycle specific capacity (**Fig. S2a**). Conversely, when the Li:Al molar ratio falls below 0.14 (corresponding to pre-lithiation capacity <0.6 mAh), irreversible capacity losses increase during Li⁺ intercalation into the Al matrix, resulting in diminished Coulombic efficiency (**Fig. S2b**). Based on these findings, we identified a Li:Al molar ratio of 0.14:1 as the optimal balance for maximizing performance.


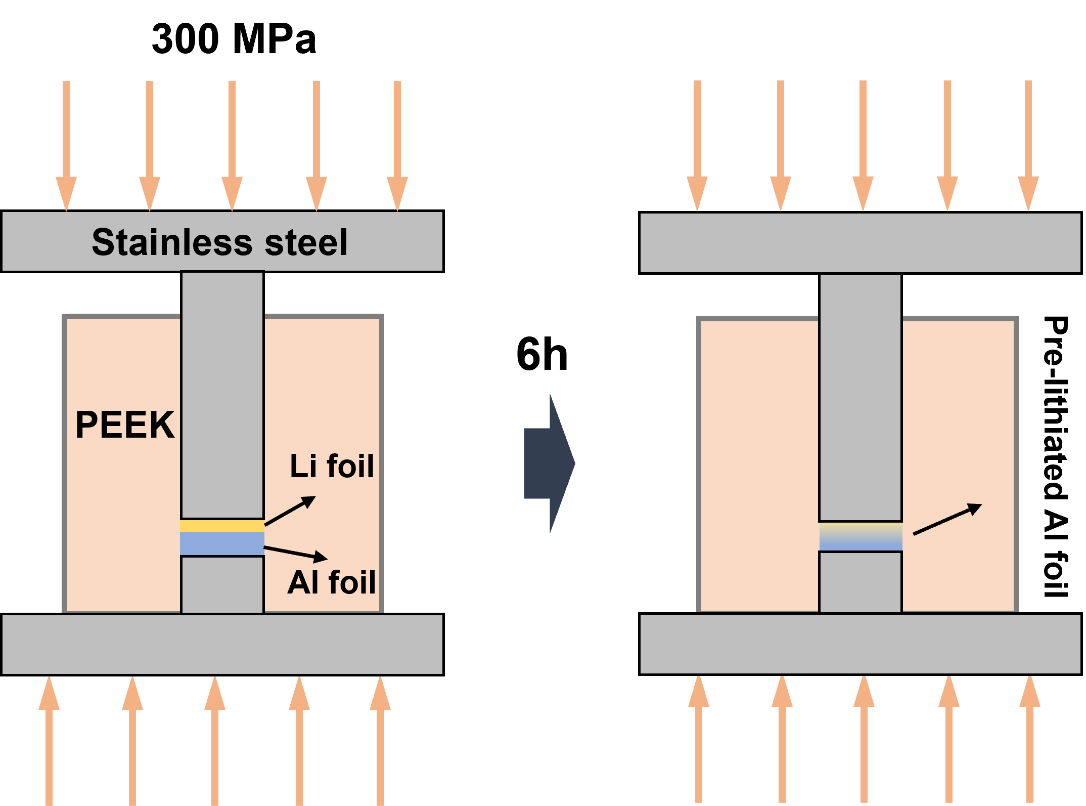


**Fig. S3** Schematic preparation process of the pre-lithiated Al anode


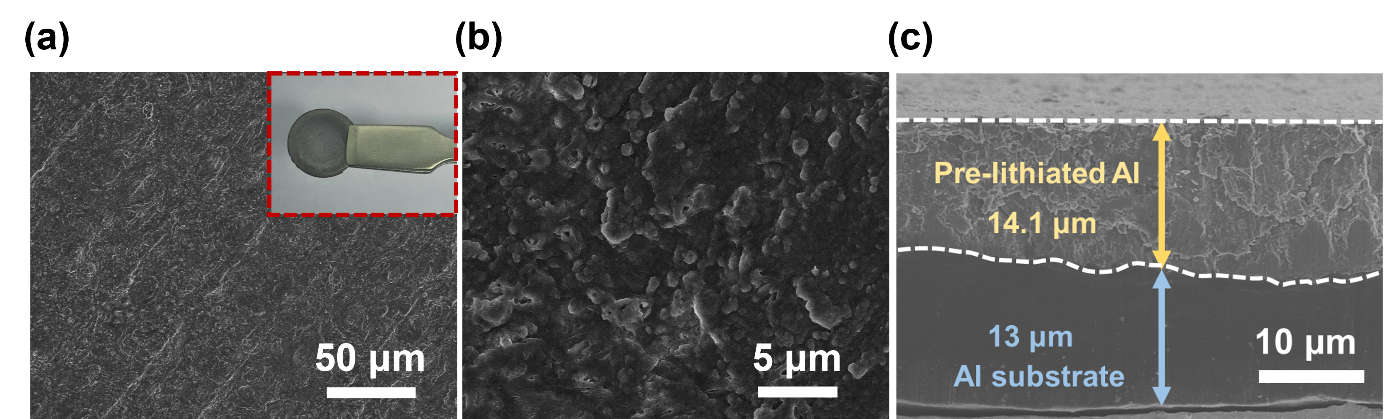


**Fig. S4** **a, b)** Surface SEM images (Inset: optical image) and **c)** cross-sectional SEM image of the pre-lithiated Al foil

**Fig. S5** XRD pattern of Al foil **a)** before and **b)** after pre-lithiation


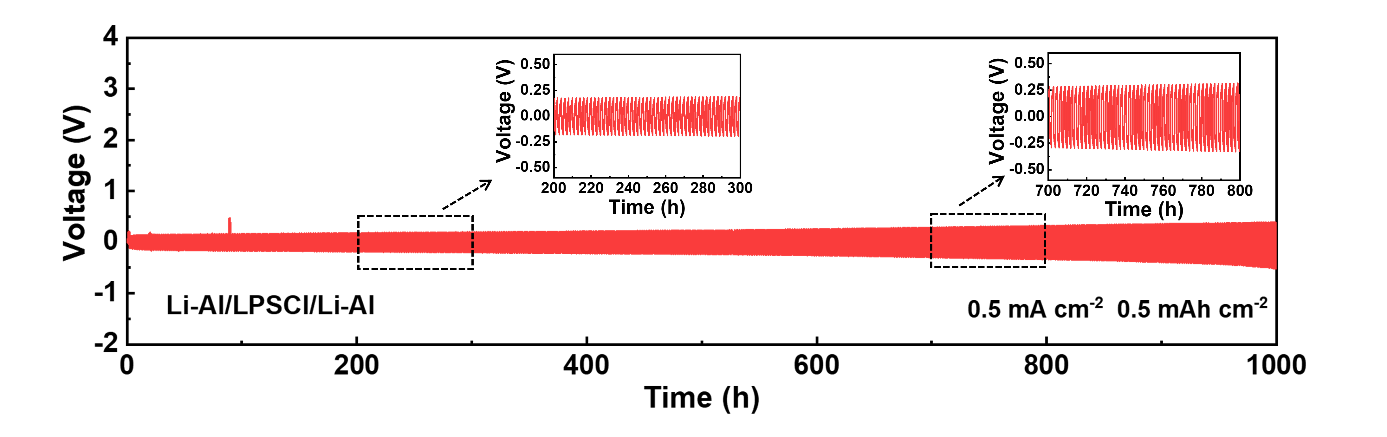


**Fig. S6** Galvanostatic Li plating/stripping profiles of the Li-Al/LPSCl/Li-Al cell at 0.5 mA cm^-2^ and 0.5 mAh cm^-2^ (Inset is the enlarged galvanostatic Li plating/stripping profiles)

**Fig. S7** Critical current density of the Li/LPSCl/Li cell

**Fig. S8** **a)** XRD pattern of the pristine NCM811 and LiNbO_3_@NCM811. **b)** SEM image and **c)** high-resolution TEM image of LiNbO_3_@NCM811


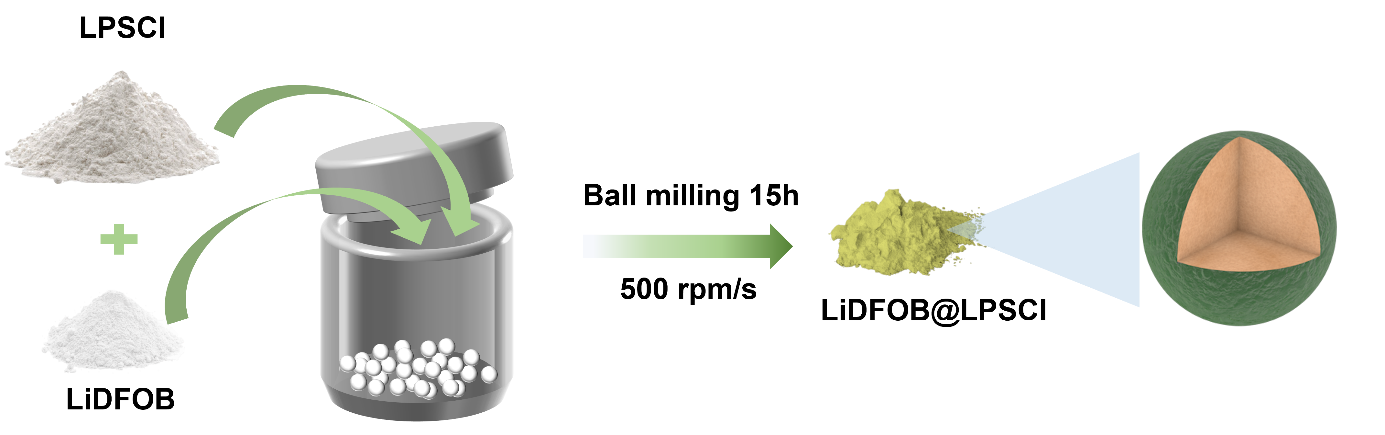


**Fig. S9** Schematic preparation process of the LiDFOB@LPSCl electrolyte

In liquid lithium-ion batteries, the inorganic constituents arising from the decomposition of lithium salts within the solid electrolyte interphase (SEI) exhibit favorable ionic conductivity coupled with electronic insulation. This unique combination effectively passivates the electrode/electrolyte interface, contributing to enhanced electrochemical stability. Drawing inspiration from this SEI-derived mechanism, we explored the potential of lithium difluoro(oxalate)borate (LiDFOB), a widely used lithium salt, as a functional additive to tailor the properties of the LPSCl solid-state electrolyte.


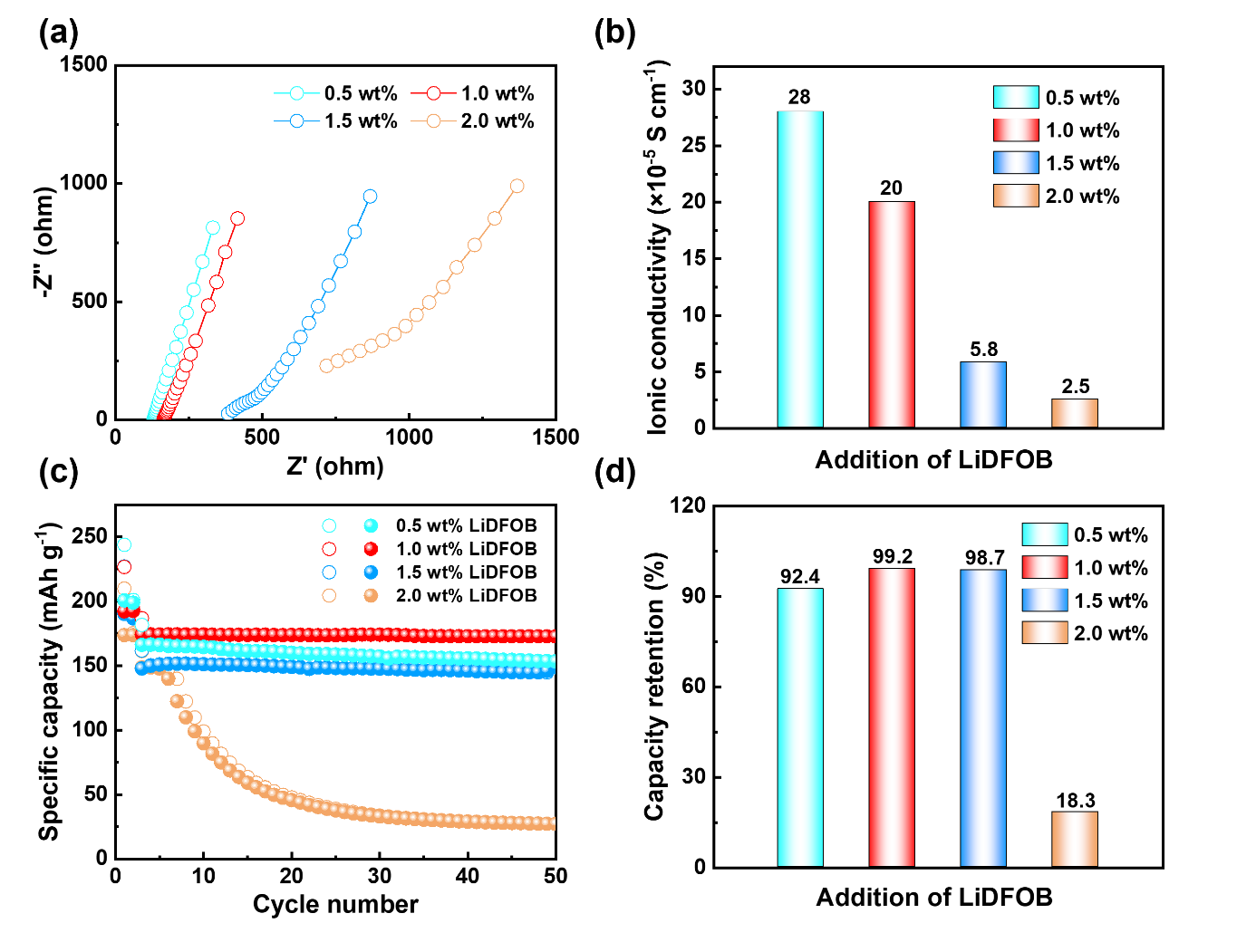


**Fig. S10** **a)** Nyquist plots of the LiDFOB@LPSCl electrolyte with different LiDFOB additions and **b)** the corresponding calculated ionic conductivities. **c)** Battery cycling performance at 0.5C with different LiDFOB additions and **d)** the corresponding capacity retentions after 50 cycles

**Fig. S11 a)** XPS full spectrum of the LiDFOB@LPSCl electrolyte, and **b-d)** corresponding F 1s, O 1s, and B 1s XPS spectra

The peaks located at 688.4 eV and 684.5 eV in F 1s spectrum correspond to the generation of Li_x_PO_y_F_z_ and LiF, respectively. In O 1s spectrum, the peaks located at 532 eV and 533.8 eV refer to the existence of Li_x_PO_y_F_z_ and LiBO_2_. And in B 1s spectrum, the peaks located at 189.2 eV and 191.1 eV can be attributed to the formation of the LiBO_2_ and B-C components.

**Fig. S12** CV test for the LiDFOB@LPSCl electrolyte to evaluate its stability at high potentials

**Fig. S13** Charge/discharge curves of the M-cathode/LiIn battery at various rates ranging from 0.1 to 2.0 C


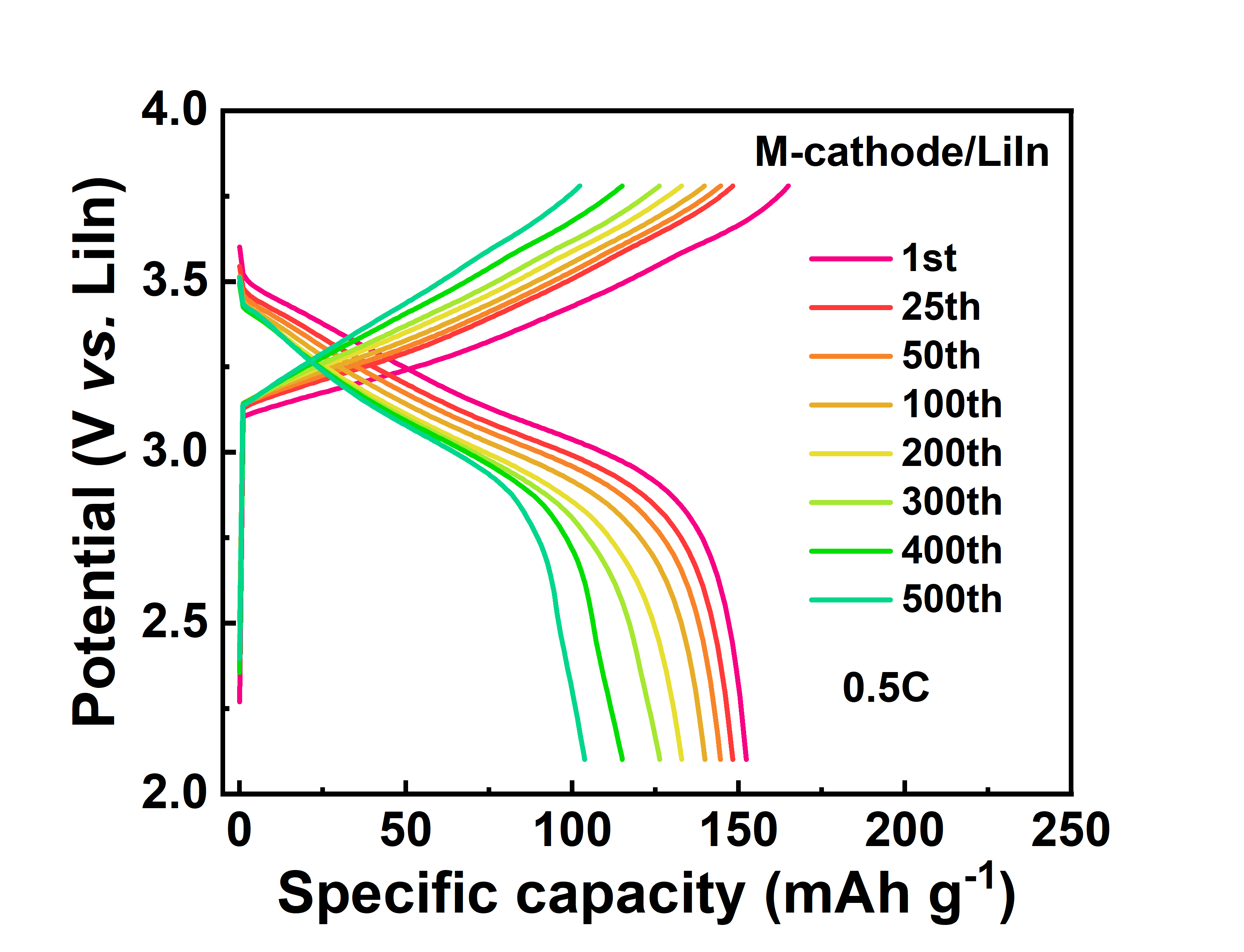


**Fig. S14** Charge/discharge curves of the M-cathode/LiIn battery at different cycles

**Fig. S15** Nyquist plots of the M-cathode/LiIn and D-cathode/LiIn batteries **a)** before and **b)** after cycling for 100 times

**Fig. S16** ToF-SIMS depth profiles of **a)** LiF^-^ and **b)** SO^-^ secondary ions over 800 s etching time for the M-cathode and D-cathode after cycling for 100 cycles


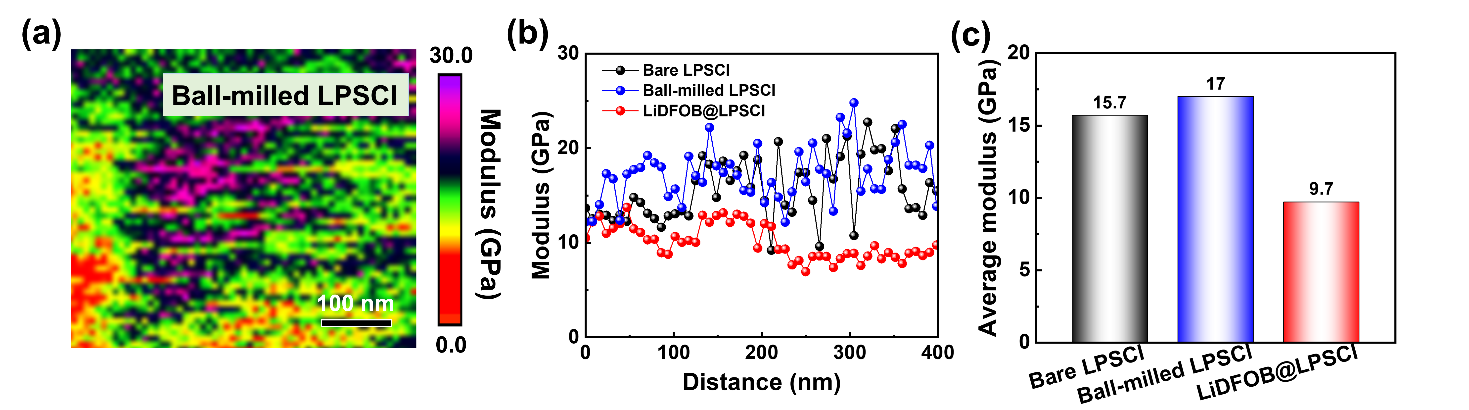


**Fig. S17** **a)** Areal distribution of Young’s modulus of the LPSCl electrolyte with ball milling treatment. **b)** Linear distribution of Young’s modulus and **c)** average modulus of the bare LPSCl, ball-milled LPSCl and LiDFOB@LPSCl electrolytes.

To investigate the impact of the ball-milling process, we performed atomic force microscopy tests on the LPSCl electrolyte with ball milling treatment but without the addition of the LiDFOB. As shown in **Fig. S17**, the ball-milled LPSCl electrolyte presented a higher average Young's modulus, which can be attributed to the refinement of the electrolyte particles under the effect of ball milling and the increase of the particle-to-particle contact area, which enhances the overall rigidity of the electrolyte.


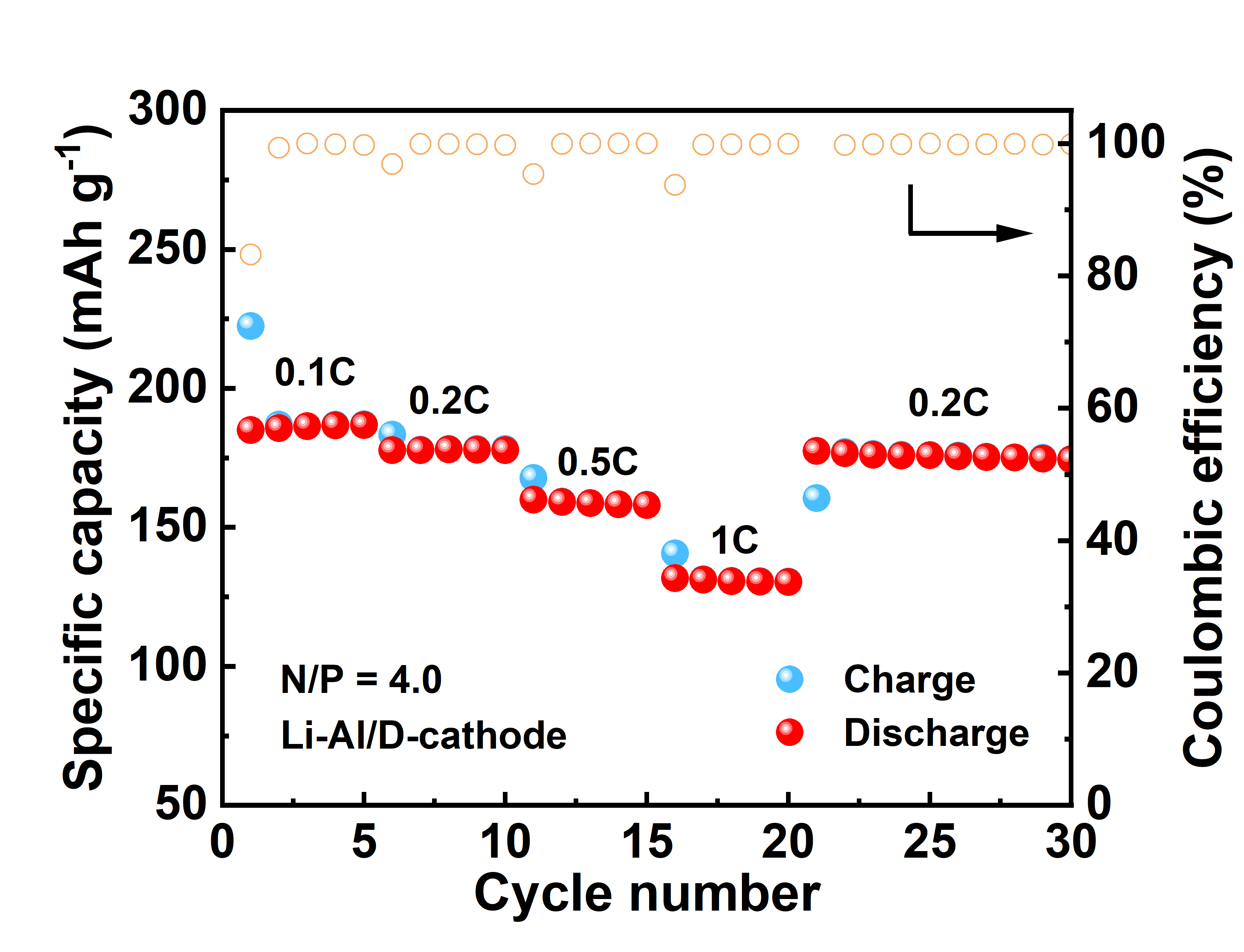


**Fig. S18** Rate performance of the Li-Al/D-cathode battery at various rates ranging from 0.1 to 1.0 C


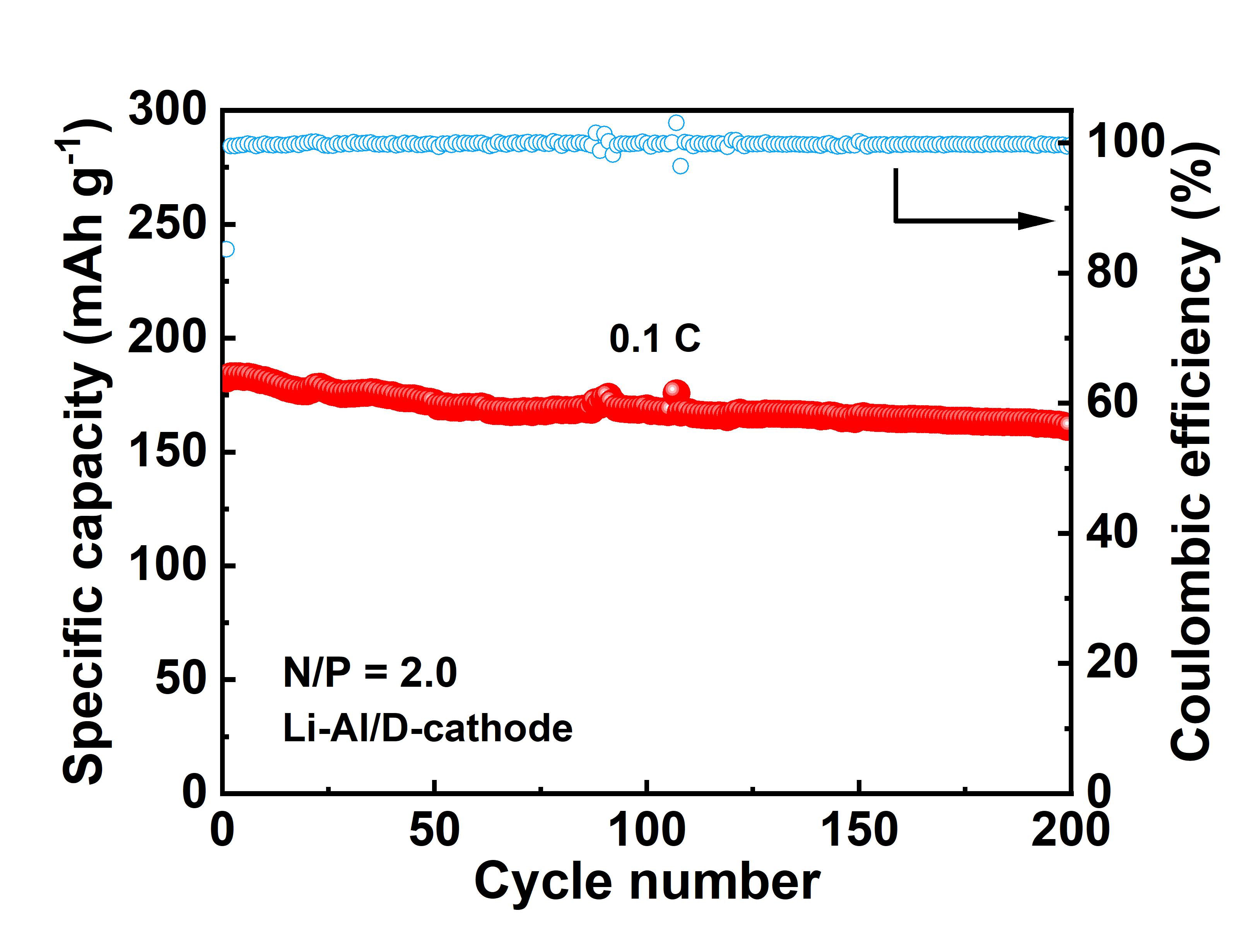


**Fig. S19** Cycling performance of the Li-Al/D-cathode battery at 0.1C with a N/P ratio of 2.0


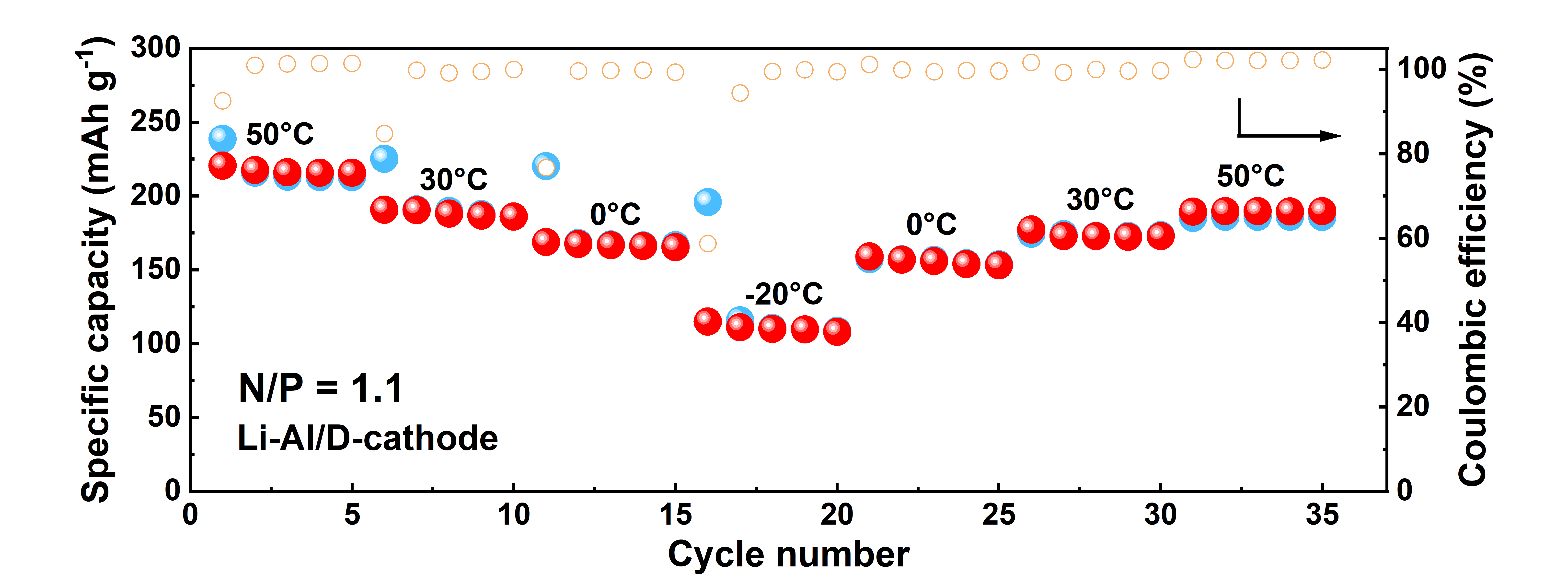


**Fig. S20** Rate performance of the Li-Al/D-cathode battery at different temperatures ranging from -20 to 50 °C
